# The functional *ALDH2* polymorphism is associated with breast cancer risk: A pooled analysis from the Breast Cancer Association Consortium

**DOI:** 10.1002/mgg3.707

**Published:** 2019-05-07

**Authors:** Tomotaka Ugai, Roger L. Milne, Hidemi Ito, Kristan J. Aronson, Manjeet K. Bolla, Tsun Chan, Ching W. Chan, Ji‐Yeob Choi, Don M. Conroy, Joe Dennis, Alison M. Dunning, Douglas F. Easton, Valerie Gaborieau, Anna Gonzalez‐Neira, Mikael Hartman, Catherine S. Healey, Motoki Iwasaki, Esther M. John, Daehee Kang, Sung‐Won Kim, Ava Kwong, Artitaya Lophatananon, Kyriaki Michailidou, Nur Aishah Mohd Taib, Kenneth Muir, Sue K. Park, Paul D. P. Pharoah, Suleeporn Sangrajrang, Chen‐Yang Shen, Xiao‐Ou Shu, John J. Spinelli, Soo H. Teo, Daniel C. Tessier, Chiu‐Chen Tseng, Shoichiro Tsugane, Daniel Vincent, Qin Wang, Anna H. Wu, Pei‐Ei Wu, Wei Zheng, Keitaro Matsuo

**Affiliations:** ^1^ Division of Cancer Epidemiology and Prevention, Department of Preventive Medicine Aichi Cancer Center Research Institute Nagoya Japan; ^2^ Cancer Epidemiology & Intelligence Division Melbourne VIC Australia; ^3^ Centre for Epidemiology and Biostatistics, School of Population and Global Health The University of Melbourne Melbourne VIC Australia; ^4^ Division of Cancer Information and Control, Department of Preventive Medicine Aichi Cancer Center Research Institute Nagoya Japan; ^5^ Department of Epidemiology Nagoya University Graduate School of Medicine Nagoya Japan; ^6^ Department of Public Health Sciences Queen's Cancer Institute, Queen's University Kingston Ontario Canada; ^7^ Centre for Cancer Genetic Epidemiology, Department of Public Health and Primary Care University of Cambridge Cambridge UK; ^8^ Hong Kong Hereditary Breast Cancer Family Registry Happy Valley Hong Kong; ^9^ Department of Pathology Hong Kong Sanatorium and Hospital Happy Valley Hong Kong; ^10^ Department of Surgery National University Health System Singapore; ^11^ Department of Biomedical Sciences Seoul National University College of Medicine Seoul Korea; ^12^ Cancer Research Institute, Seoul National University College of Medicine Seoul Korea; ^13^ Centre for Cancer Genetic Epidemiology, Department of Oncology University of Cambridge Cambridge UK; ^14^ Genetic Epidemiology Group, International Agency for Research on Cancer Lyon France; ^15^ Human Cancer Genetics Program Spanish National Cancer Research Centre Madrid Spain; ^16^ Saw Swee Hock School of Public Health National University of Singapore Singapore; ^17^ Epidemiology and Prevention Group, Center for Public Health Sciences National Cancer Center Tokyo Japan; ^18^ Department of Medicine and Stanford Cancer Institute Stanford University School of Medicine Stanford California USA; ^19^ Department of Preventive Medicine Seoul National University College of Medicine, Seoul National University Seoul Korea; ^20^ Department of Surgery Daerim Saint Mary's Hospital Seoul Korea; ^21^ Department of Surgery Queen Mary Hospital, The University of Hong Kong Happy Valley Hong Kong; ^22^ Department of Surgery Hong Kong Sanatorium and Hospital Happy Valley Hong Kong; ^23^ Division of Health Sciences, Warwick Medical School Warwick University Coventry UK; ^24^ Division of Population Sciences, Warwick Medical School Warwick University Coventry UK; ^25^ Department of Electron Microscopy/Molecular Pathology The Cyprus Institute of Neurology and Genetics Nicosia Cyprus; ^26^ Breast Cancer Research Unit University Malaya Cancer Research Institute, University Malaya Medical Centre Kuala Lumpur Malaysia; ^27^ Department of Preventive Medicine Seoul National University College of Medicine Seoul Korea; ^28^ National Cancer Institute Bangkok Thailand; ^29^ Taiwan Biobank Institute of Biomedical Sciences, Academia Sinica Taipei Taiwan; ^30^ College of Public Health China Medical University Taichong Taiwan; ^31^ Division of Epidemiology, Department of Medicine Vanderbilt Epidemiology Center, Vanderbilt‐Ingram Cancer Center, Vanderbilt University School of Medicine Nashville Tennessee USA; ^32^ School of Population & Public Health University of British Columbia Vancouver British Columbia Canada; ^33^ Cancer Control Research, BC Cancer Agency Vancouver British Columbia Canada; ^34^ Cancer Research Initiatives Foundation Sime Darby Medical Centre Subang Jaya Malaysia; ^35^ McGill University and Génome Québec Innovation Centre Montreal Quebec Canada; ^36^ Department of Preventive Medicine, Keck School of Medicine University of Southern California Los Angeles California USA

**Keywords:** acetaldehyde, alcohol drinking, aldehyde dehydrogenase‐2, breast cancer, single nucleotide polymorphism

## Abstract

**Background:**

Epidemiological studies consistently indicate that alcohol consumption is an independent risk factor for female breast cancer (BC). Although the aldehyde dehydrogenase 2 (*ALDH2*) polymorphism (rs671: Glu>Lys) has a strong effect on acetaldehyde metabolism, the association of rs671 with BC risk and its interaction with alcohol intake have not been fully elucidated. We conducted a pooled analysis of 14 case‐control studies, with individual data on Asian ancestry women participating in the Breast Cancer Association Consortium.

**Methods:**

We included 12,595 invasive BC cases and 12,884 controls for the analysis of rs671 and BC risk, and 2,849 invasive BC cases and 3,680 controls for the analysis of the gene‐environment interaction between rs671 and alcohol intake for BC risk. The pooled odds ratios (OR) with 95% confidence intervals (CI) associated with rs671 and its interaction with alcohol intake for BC risk were estimated using logistic regression models.

**Results:**

The Lys/Lys genotype of rs671 was associated with increased BC risk (OR = 1.16, 95% CI 1.03–1.30, *p* = 0.014). According to tumor characteristics, the Lys/Lys genotype was associated with estrogen receptor (ER)‐positive BC (OR = 1.19, 95% CI 1.05–1.36, *p* = 0.008), progesterone receptor (PR)‐positive BC (OR = 1.19, 95% CI 1.03–1.36, *p* = 0.015), and human epidermal growth factor receptor 2 (HER2)‐negative BC (OR = 1.25, 95% CI 1.05–1.48, *p* = 0.012). No evidence of a gene‐environment interaction was observed between rs671 and alcohol intake (*p* = 0.537).

**Conclusion:**

This study suggests that the Lys/Lys genotype confers susceptibility to BC risk among women of Asian ancestry, particularly for ER‐positive, PR‐positive, and HER2‐negative tumor types.

## 1 INTRODUCTION

Epidemiological studies consistently indicate that alcohol is an independent risk factor for female breast cancer (BC) (Singletary & Gapstur, 2001). The International Agency for Research on Cancer concluded that there is sufficient evidence to classify alcohol as a carcinogen for female BC (IARC Working Group on the Evaluation of Carcinogenic Risks to Humans, 2010). One hypothesized mechanism behind alcohol‐related breast carcinogenesis is the involvement of acetaldehyde, a metabolite of ethanol. An impact of acetaldehyde on carcinogenesis for several types of alcohol‐induced cancers has been shown in experimental models (Brooks & Theruvathu, 2005). Molecular epidemiological studies demonstrated a gene‐environment interaction between a functional aldehyde dehydrogenase 2 (*ALDH2*) polymorphism (rs671: Glu>Lys, OMIM: 100650) and alcohol intake for esophageal and upper digestive tract cancers in East Asian countries (Matsuo et al., 2001; Oze et al., 2010), where rs671 is prevalent (Li et al., 2009). These studies support the hypothesis that acetaldehyde is a carcinogen. The Glu/Lys heterozygotes of rs671 have far less than half of ALDH2 activity of Glu/Glu homozygotes, and the Lys/Lys homozygotes have no detectable ALDH2 activity, which leads to high acetaldehyde concentrations upon alcohol intake in individuals harboring the Lys allele (Crabb, Edenberg, Bosron, & Li, 1989). Therefore, exploring the association of rs671 with BC risk and its interaction with alcohol intake is one approach to elucidate whether acetaldehyde is a causative agent for breast carcinogenesis. To date, evidence of an association of rs671 with BC risk is scarce; statistically significant associations have not been observed in case‐control studies in Japan (456 cases and 912 controls) (Kawase et al., 2009) Korea (346 cases and 377 controls) (Choi et al., 2003) or Thailand (561 cases and 486 controls) (Sangrajrang et al., 2010). We conducted a pooled analysis of individual genetic and alcohol consumption data for women of Asian ancestry participating in studies in the Breast Cancer Association Consortium (BCAC) with at least 18 times larger sample size than previous studies.

## 2 METHODS

### 2.1 Study population

We used data from 14 case‐control studies in the BCAC. Table 1 shows participating studies contributing to this pooled analysis. All study participants were of Asian ancestry and recruited from studies conducted in Asian countries, Canada, and the USA. Eight studies were hospital‐based, five were population‐based, and one included hospital‐based cases and population‐based controls. We included 12,595 BC cases and 12,884 controls for the analysis of rs671 and BC risk. For the analysis of the gene‐environment interaction between rs671 and alcohol intake for BC risk, we included 2,849 BC cases and 3,680 controls after excluding participants with missing values for alcohol intake from seven studies. All studies were approved by their local ethics review boards, and all participants provided informed consent. This investigation was approved by a human research investigations committee at Aichi Cancer Center.

**Table 1:** List of participating studies and number of participants

Study acronym	Study name	Study design	Country	Subjects of analysis for rs671				Subjects of analysis for GE interaction			
				Case	Control	Lys allele frequency among cases (%)	Lys allele frequency among controls (%)	Case	Control	Lys allele frequency among cases (%)	Lys allele frequency among controls (%)
ACP	Asia Cancer Program	Hospital based case‐control study	Thailand	830	1,060	8.9	8.0	—	—	—	—
CBCS	Canadian Breast Cancer Study	Population‐based case‐control study	Canada	252	170	28.6	20.0	—	—	—	—
HERPACC	Hospital‐based Epidemiologic Research Program at Aichi Cancer	Hospital‐based case‐control study	Japan	792	1,659	29.9	28.3	783	1,632	30.1	28.6
HKBCS	Hong Kong Breast Cancer Study	Hospital‐based case‐control study	China	466	451	32.1	28.4	—	—	—	—
KOHBRA	Korean Hereditary Breast Cancer Study	Population‐based case‐control study	Korea	1,251	665	17.1	15.9	413	601	6.8	15.4
LAABC	Los Angeles County Asian‐American Breast Cancer Case‐Control Study	Population‐based case‐control study	USA	808	990	24.9	27.5	808	990	24.9	27.5
MYBRCA	Malaysian Breast Cancer Genetic Study	Hospital‐based case‐control study	Malaysia	1,408	1,866	24.5	22.6	—	—	—	—
NC‐BCFR	Northern California Breast Cancer Family Registry	Population‐based case‐control study	USA	446	52	21.4	21.2	400	46	22.4	23.9
NGOBCS	Nagano Breast Cancer Study	Hospital‐based case‐control study	Japan	366	366	25.4	23.6	366	365	25.4	23.7
SBCGS	Shanghai Breast Cancer Genetic Study	Population‐based case‐control study, cohort study	China	1,644	1,827	24.9	23.9	5	46	10.0	7.6
SEBCS	Seoul Breast Cancer Study	Hospital‐based case‐control study	Korea	2,129	2,236	16.9	15.1	74	—	2.7	—
SGBCC	Singapore Breast Cancer Cohort	Hospital based breast cancer cohort and population‐based controls	Singapore	775	798	20.8	23.6	—	—	—	—
TBCS	IARC‐Thai Breast Cancer Study	Hospital‐based case‐control study	Thailand	138	253	6.9	11.7	—	—	—	—
TWBCS	Taiwanese Breast Cancer Study	Hospital‐based case‐control study	Taiwan	1,290	491	27.8	31.5	—	—	—	—
Total				12,595	12,884	22.1	21.4	2,849	3,680	22.8	25.3

Abbreviation: GE interaction, gene‐environment interaction.

### 2.2 Genotyping methods

Genotyping was carried out using the iCOGS array (http://ccge.medschl.cam.ac.uk/research/consortia/icogs/), or the OncoArray (https://support.illumina.com/downloads/infinium-oncoarray-500k-v1-0-product-files.html). Details of array design, genotyping, postgenotyping quality control, and imputation have been provided elsewhere (Michailidou et al., 2013, 2017). The rs671 SNP on *ALDH2* was a candidate SNP selected on the basis of specific hypotheses described above.

To adjust for potential population stratification, principal components analyses (PCA) were carried out separately for Asian subgroups. Briefly, PCA was performed based on a subset of 37,000 uncorrelated SNPs for the iCOGS data and based on 33,661 uncorrelated SNPs for the OncoArray data. For the present analyses, we used two Asian principal components for the iCOGS dataset and 10 Asian principal components for the OncoArray dataset as covariates. Further details have been provided in previous articles (Michailidou et al., 2013, 2017).

### 2.3 Alcohol assessment

Each study ascertained alcohol intake via self‐reported questionnaire. Daily alcohol intake in grams was determined by summing the product of frequency of consumption of specified alcoholic beverages (beer, wine, and other alcoholic beverages) by the alcohol content of each beverage using national estimates of alcohol content for that country. The exposure period was the year preceding recruitment. A multistep harmonization procedure was used to reconcile differences in individual study questionnaires.

### 2.4 Statistical analysis

To assess the associations of rs671 with BC risk, we estimated odds ratios (ORs) with 95% confidence intervals (CIs) by unconditional logistic regression models using the Glu/Glu genotype as reference. This was done separately for iCOGS and OncoArray datasets, and results were combined by a fixed‐effects meta‐analysis. The ORs were adjusted for age, Asian principal components, and study. We also evaluated the associations by tumor characteristics (estrogen receptor, ER; progesterone receptor, PR; human epidermal growth factor receptor 2, HER2) and tumor subtypes (luminal [either ER or PR positive, HER2 negative], triple positive [ER, PR, HER2 positive], HER2 enrich [ER, PR negative, HER2 positive], triple negative [ER, PR, HER2 negative]) using cases with these specific characteristics. Heterogeneity by tumor characteristics and between studies was assessed using Cochran's Q test. We assessed the gene‐environment interaction between rs671 and alcohol intake by including an interaction term. Alcohol intake was classified in three ways: 1) two categories (none, any alcohol intake); 2) three categories (none, <15 g ethanol/day, ≥15 g ethanol/day); and 3) four categories (none, <15 g ethanol/day, 15–30 g ethanol/day, ≥30 g ethanol/day). We also performed stratified analyses by menopausal status: women with missing menopausal status were considered premenopausal if they were ≤50 years or postmenopausal if >50 years. All statistical analyses were performed using Stata version 15.1 (Stata Corp., College Station, TX, USA), with a P value <0.05 considered to be statistically significant.

## 3 RESULTS

Demographic characteristics of participants are shown in Table 2. The median age was 50 years for both cases and controls, with a higher proportion of women in the oldest age groups for cases. The proportion of nondrinkers and heavy drinkers (≥15 g ethanol/day) was higher among controls than cases, possibly due to the smaller number of unknown category in controls (71.4%) than in cases (77.4%). The distributions of tumor characteristics among cases were 7,648 ER positive (60.7%), 6,308 PR positive (50.1%), and 3,054 HER2 positive (24.3%) for participants included in the analysis of rs671 alone and, 1,871 ER positive (65.7%), 1,620 PR positive (56.9%), and 552 HER2 positive (19.4%) for those in the analysis of gene‐environment interaction, respectively.

**Table 2:** Characteristics of cases and controls

	Subjects of analysis for rs671				Subjects of analysis for GE interaction			
	Cases (*N* = 12,595)	(%)	Control (*N* = 12,884)	(%)	Cases (*N* = 2,849)	(%)	Control (*N* = 3,680)	(%)
Age (years)								
Median (range)	50 (20–91)		50 (15–92)		50 (20–81)		50 (19–86)	
≦29	205	1.6	300	2.3	60	2.1	68	1.9
30–39	1,641	13.0	1,254	9.7	421	14.8	408	11.1
40–49	4,255	33.8	4,547	35.3	845	29.7	1,234	33.5
50–59	3,847	30.5	4,179	32.4	830	29.1	1,117	30.4
60–69	1,911	15.2	2,138	16.6	498	17.5	638	17.3
≧70	736	5.8	466	3.6	195	6.8	215	5.8
Alcohol consumption†								
g/day (mean ± *SD*)	31.2 ± 91.2		30.5 ± 83.0		31.2 ± 91.2		30.5 ± 83.0	
Nondrinker	1,746	13.9	2,348	18.2	1,746	61.3	2,348	63.8
<15 g ethanol/day	895	7.1	1,052	8.2	895	31.4	1,052	28.6
≥15 g ethanol/day	208	1.7	280	2.2	208	7.3	280	7.6
Unknown	9,746	77.4	9,204	71.4				
ALDH2 Glu/Glu genotype								
g/day (mean ± *SD*)	42.4 ± 103.9		44.8 ± 102.0		42.4 ± 103.9		44.8 ± 102.0	
Nondrinker	828	10.6	1,040	12.9	828	48.0	1,040	50.7
<15 g ethanol/day	719	9.2	774	9.6	719	41.7	774	37.8
≥15 g ethanol/day	178	2.3	236	2.9	178	10.	236	11.5
Unknown	6,056	77.8	5,988	74.5				
ALDH2 Glu/Lys genotype								
g/day (mean ± *SD*)	16.1 ± 68.5		13.3 ± 43.0		16.1 ± 68.5		13.3 ± 43.0	
Nondrinker	745	18.3	1,076	25.8	745	78.6	1,076	77.1
<15 g ethanol/day	173	4.3	276	6.6	173	18.3	276	19.8
≥15 g ethanol/day	30	0.7	44	1.1	30	3.2	44	3.2
Unknown	3,122	76.7	2,779	66.6				
ALDH2 Lys/Lys genotype								
g/day (mean ± *SD*)	0.4 ± 3.4		0.5 ± 5.1		0.4 ± 3.4		0.5 ± 5.1	
Nondrinker	232	99.2	173	98.3	232	99.2	173	98.3
<15 g ethanol/day	2	0.8	3	1.7	2	0.8	3	1.7
≥15 g ethanol/day	0	0	0	0	0	0	0	0
Unknown	0	0	0	0				
Menopausal status								
Premenopausal	3,690	29.3	5,234	40.6	836	29.3	1,393	37.9
Postmenopausal	4,287	34.0	4,830	37.5	879	30.9	1,246	33.9
Unknown	4,618	36.7	2,820	21.9	1,134	39.8	1,041	28.3
ER status								
Positive	7,648	60.7			1,871	65.7		
Negative	3,701	29.4			658	23.1		
Unknown	1,246	9.9			320	11.2		
PR status								
Positive	6,308	50.1			1,620	56.9		
Negative	3,776	30.0			865	30.4		
Unknown	2,511	19.9			364	12.8		
HER2 status								
Positive	3,054	24.3			552	19.4		
Negative	4,054	32.2			557	19.6		
Unknown	5,487	43.6			1,740	61.1		

Abbreviations: ALDH2, aldehyde dehydrogenase 2; ER, estrogen receptor; GE interaction, gene‐environment interaction; HER2, human epidermal growth factor receptor 2; PR, progesterone receptor.

† Exposure period was the year preceding recruitment.

Table 3 presents the associations of rs671 with BC risk. Overall, the Lys/Lys genotype was associated with increased BC risk, with OR of 1.16 (95% CI = 1.03–1.30, *p = *0.014) relative to Glu/Glu genotype. According to tumor characteristics, we observed an association of the Lys/Lys genotype with ER‐positive BC (OR = 1.19, 95% CI 1.05–1.36, *p* = 0.008), PR‐positive BC (OR = 1.19, 95% CI 1.03–1.36, *p* = 0.015), and HER2‐negative BC (OR = 1.25, 95% CI 1.05–1.48, *p* = 0.012), but not with ER‐negative BC (OR = 1.07, 95% CI 0.90–1.27, *p* = 0.453), PR‐negative BC (OR = 1.13, 95% CI 0.95–1.34, *p* = 0.176), or HER2‐positive BC (OR = 1.19, 95% CI 0.97–1.48, *p* = 0.102), although no statistically significant heterogeneity was observed by tumor characteristics. According to tumor subtypes, the Lys/Lys genotype was only associated with luminal BC (OR = 1.30, 95% CI 1.09–1.55, *p* = 0.004), and not with other subtypes (Table 4). No evidence of heterogeneity was also observed by menopausal status (Table S1).

**Table 3:** Association between ALDH2 genotype and breast cancer risk

	ALDH2 genotype			*p* for heterogeneity between tumor characteristics	
	Glu/Glu	Glu/Lys	Lys/Lys	For Glu/Lys	For Lys/Lys
Overall					
Cases/controls	7,781/8,038	4,070/4,175	744/671		
OR (95% CI)†	1 (ref.)	1.03 (0.97–1.08, *p* = 0.350)	1.16 (1.03–1.30, *p* = 0.014)		
ER status					
Positive					
Cases/controls	4,636/8,038	2,531/4,175	481/671		
OR (95% CI)†	1 (ref.)	1.01 (0.95–1.08, *p* = 0.669)	1.19 (1.05–1.36, *p* = 0.008)	0.447	0.329
Negative					
Cases/control	2,321/8,038	1,187/4,175	193/671		
OR (95% CI)†	1 (ref.)	1.05 (0.97–1.14, *p* = 0.257)	1.07 (0.90–1.27, *p* = 0.453)		
PR status					
Positive					
Cases/controls	3,842/8,038	2,066/4,175	400/671		
OR (95% CI)†	1 (ref.)	0.98 (0.92–1.05, *p* = 0.591)	1.19 (1.03–1.36, *p* = 0.015)	0.410	0.653
Negative					
Cases/control	2,333/8,038	1,238/4,175	205/671		
OR (95% CI)†	1 (ref.)	1.02 (0.95–1.11, *p* = 0.545)	1.13 (0.95–1.34, *p* = 0.176)		
HER2 status					
Positive					
Cases/control	1,961/8,038	940/4,175	153/671		
OR (95% CI)†	1 (ref.)	1.02 (0.92–1.14, *p* = 0.674)	1.19 (0.97–1.48, *p* = 0.102)	1.000	0.720
Negative					
Cases/control	2,521/7,841	1,287/4,175	246/671		
OR (95% CI)†	1 (ref.)	1.02 (0.93–1.11, *p* = 0.722)	1.25 (1.05–1.48, *p* = 0.012)		

Abbreviations: ALDH2, aldehyde dehydrogenase 2; CI, confidence intervals; ER, estrogen receptor; HER2, human epidermal growth factor receptor 2; OR, odds ratios; PR, progesterone receptor.

† ORs were adjusted for age (continuous), Asian principal components and study site.

**Table 4:** Association between ALDH2 genotype and breast cancer risk by tumor subtypes

	ALDH2 genotype			*p* for heterogeneity between tumor characteristics	
	Glu/Glu	Glu/Lys	Lys/Lys	For Glu/Lys	For Lys/Lys
Luminal					
Cases/controls	1,950/8,038	979/4,175	198/671		
OR (95% CI)†	1 (ref.)	1.00 (0.92–1.10, *p* = 0.916)	1.30 (1.09–1.55, *p* = 0.004)	0.452	0.755
Triple positive					
Cases/controls	1,202/8,038	583/4,175	93/671		
OR (95% CI)†	1 (ref.)	1.03 (0.91–1.16, *p* = 0.640)	1.19 (0.93–1.53, *p* = 0.164)		
HER2 enrich					
Cases/controls	694/8,038	322/4,175	55/671		
OR (95% CI)†	1 (ref.)	0.96 (0.83–1.11, *p* = 0.557)	1.12 (0.83–1.51, *p* = 0.453)		
Triple negative					
Cases/control	546/8,038	310/4,175	46/671		
OR (95% CI)†	1 (ref.)	1.13 (0.97–1.32, *p* = 0.108)	1.11 (0.81–1.53, *p* = 0.519)		

Abbreviations: ALDH2, aldehyde dehydrogenase 2; CI, confidence intervals; HER2, human epidermal growth factor receptor 2; OR, odds ratios.

† ORs were adjusted for age (continuous), Asian principal components and study site.

Figure S1 and Figure S2 show the forest plots of study‐specific ORs for the association between rs671 and BC risk. With regard to the association between the Glu/Lys genotype and BC risk, there was no evidence of between‐study heterogeneity (*p* for heterogeneity = 0.380). In contrast, significant between‐study heterogeneity was observed for the association of the Lys/Lys genotype with BC risk (*p* for heterogeneity = 0.003), which was mainly attributable to a strong positive association for CBCS and a strong inverse association for ACP and TWBCS. However, exclusion of these studies did not alter the significant association of the Lys/Lys genotype with BC risk (OR = 1.18, 95% CI 1.05–1.33, *p* = 0.008) and there was no longer evidence of between‐study heterogeneity (*p* for heterogeneity = 0.133). Furthermore, when we repeated analyses using random effects meta‐analyses to calculate summary study‐specific estimates, the results did not change substantially (Table S2).

Stratified analyses by alcohol intake categories assessing a gene‐environment interaction between rs671 and alcohol intake showed no evidence of interaction, although the sample size is small compared to the analysis of rs671 and BC risk (Table S3, *p* for interaction = 0.537).

## 4 DISCUSSION

In this study, we found that the Lys/Lys genotype of rs671 was associated with increased BC risk among women of Asian ancestry. No evidence of interaction was observed between rs671 and alcohol intake. This is the largest study to date to perform this evaluation quantitatively using high‐quality individual‐level data for Asian women.

Several epidemiological studies have reported a gene‐environment interaction between rs671 and alcohol intake for several types of cancer (Hiraki et al., 2007; Ishioka et al., 2018; Masaoka et al., 2016; Matsuo et al., 2001, 2013; Oze et al., 2010). Our findings are not consistent with our hypothesis of gene‐environment interaction between rs671 and alcohol intake. Considering the established impact of rs671 on cancer risk, this lack of interaction suggests that acetaldehyde may be less influential in breast carcinogenesis. Other biological mechanisms for alcohol‐related breast carcinogenesis have been hypothesized, including increased circulating estrogens and androgens, enhancement of mammary gland susceptibility to carcinogenesis, increased mammary carcinogen DNA damage, interference of folate metabolism by alcohol, and greater potential for invasiveness into BC cells (Bernstein & Ross, 1993; Singletary & Gapstur, 2001; Singletary & McNary, 1994; Stolzenberg‐Solomon et al., 2006). To better understand the etiologic nature of the effect of alcohol on breast carcinogenesis, further investigations are needed.

We observed an association of the Lys/Lys genotype with increased BC risk. Because individuals with the Lys/Lys genotype have no detectable ALDH2 activity and almost completely refrain from drinking due to severe adverse reactions caused by acetaldehyde (e.g., facial flushing, nausea and headache) (Matsuo et al., 2006), the observed genetic association suggests that the Lys/Lys genotype confers susceptibility to BC risk independently of alcohol intake. ALDH2 plays a key role in removal of not only ethanol‐derived acetaldehyde, but also other toxic endogenous aldehydes such as 4‐hydroxy‐2‐nonenal (4‐HNE) and malondialdehyde (Chen, Ferreira, Gross, & Mochly‐Rosen, 2014). These endogenous aldehydes have been reported to cause DNA damage and might be related to breast carcinogenesis (Chen et al., 2014; Garaycoechea et al., 2018). In addition, we did not find an association of the Glu/Lys genotype with BC risk. This suggest that ALDH2 activity of the Glu/Lys homozygotes may be sufficient for detoxifying toxic endogenous aldehydes related to breast carcinogenesis. In contrast, the Lys/Lys homozygotes have no detectable ALDH2 activity, thus may not tolerate these endogenous aldehydes. Furthermore, the Lys/Lys genotype was associated with increased risk only in hormone receptor positive BC, and not in hormone receptor negative BC. These results suggest that the biological mechanism could be through a hormonal receptor mediated pathway (Zhang, Man, Zhao, Dong, & Ma, 2014). The evidence of an association of rs671 with BC risk is scarce and may warrant additional evaluation in future studies.

The strengths of this investigation include the analysis of individual‐level data from a large sample of Asian women, allowing us to obtain stable, and precise summary estimates of the association of rs671 with BC risk. Other strengths are the uniform genotyping procedures and quality‐control measures undertaken for the iCOGS and the OncoArray, respectively. We were also able to control for population stratification by including Asian principal components as a covariate to control for residual genetic heterogeneity. Furthermore, the Lys allele of rs671 is only prevalent in East Asia, and has not been found in Caucasians or Africans (Li et al., 2009). Thus, this analysis is unique and can be performed only among Asian women. Several limitations also warrant consideration. First, we could not evaluate the association between alcohol intake and BC risk because there were a lot of missing data on potential confounding factors (e.g., smoking, estrogen‐related factors) and we were not able to control for them. However, genotypes are fixed at birth and these factors cannot influence genotypes; therefore, our results about rs671 and BC risk may be unbiased even though we did not adjust for these factors. Second, even though all study participants were of Asian ancestry, the heterogeneity across study populations, designs, and methods are potential limitations. Third, careful interpretation of results from the analysis of gene‐environment interaction and stratified analyses is necessary because we had a limited number of participants in some sub‐groups and did not adjust for multiple comparisons.

In conclusion, we observed an association between the Lys/Lys genotype of rs671 and increased BC risk. Among women of Asian ancestry, this study suggests that the Lys/Lys genotype confers susceptibility to BC risk, particularly for ER‐positive, PR‐positive, and HER2‐negative tumor types. These findings warrant further investigation in future studies.

## CONFLICT OF INTEREST

The authors declare no conflict of interest.
