# Supplementary figures and images for: The functional ALDH2 polymorphism is associated with breast cancer risk: A pooled analysis from the Breast Cancer Association Consortium
Source: Mol Genet Genomic Med. 2019 May 7;7(6):e707. doi: 10.1002/mgg3.707 (PMC6565553; doi:10.1002/mgg3.707)

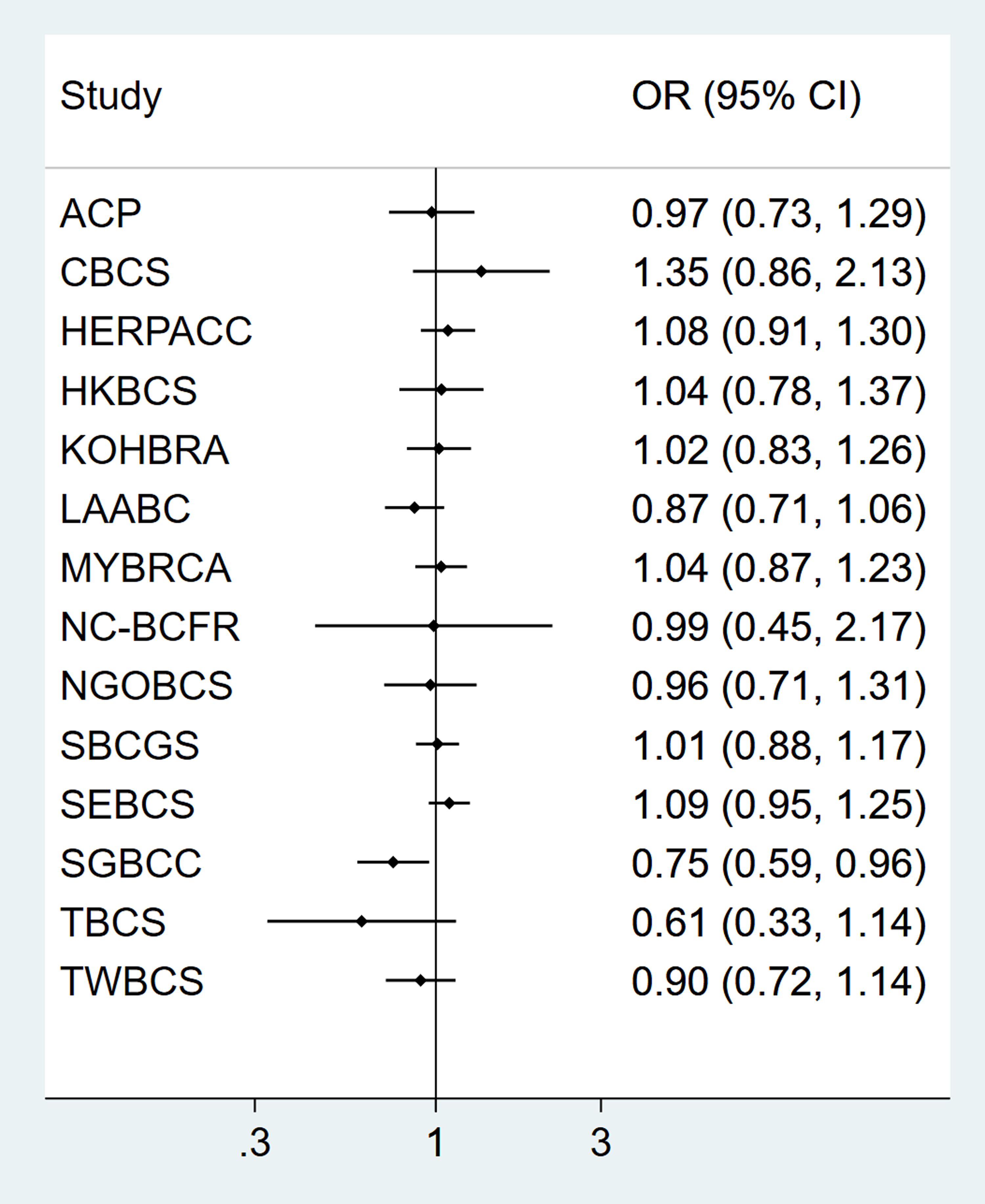

Supplement: Supplementary file 1 [file MGG3-7-e707-s001.tif]

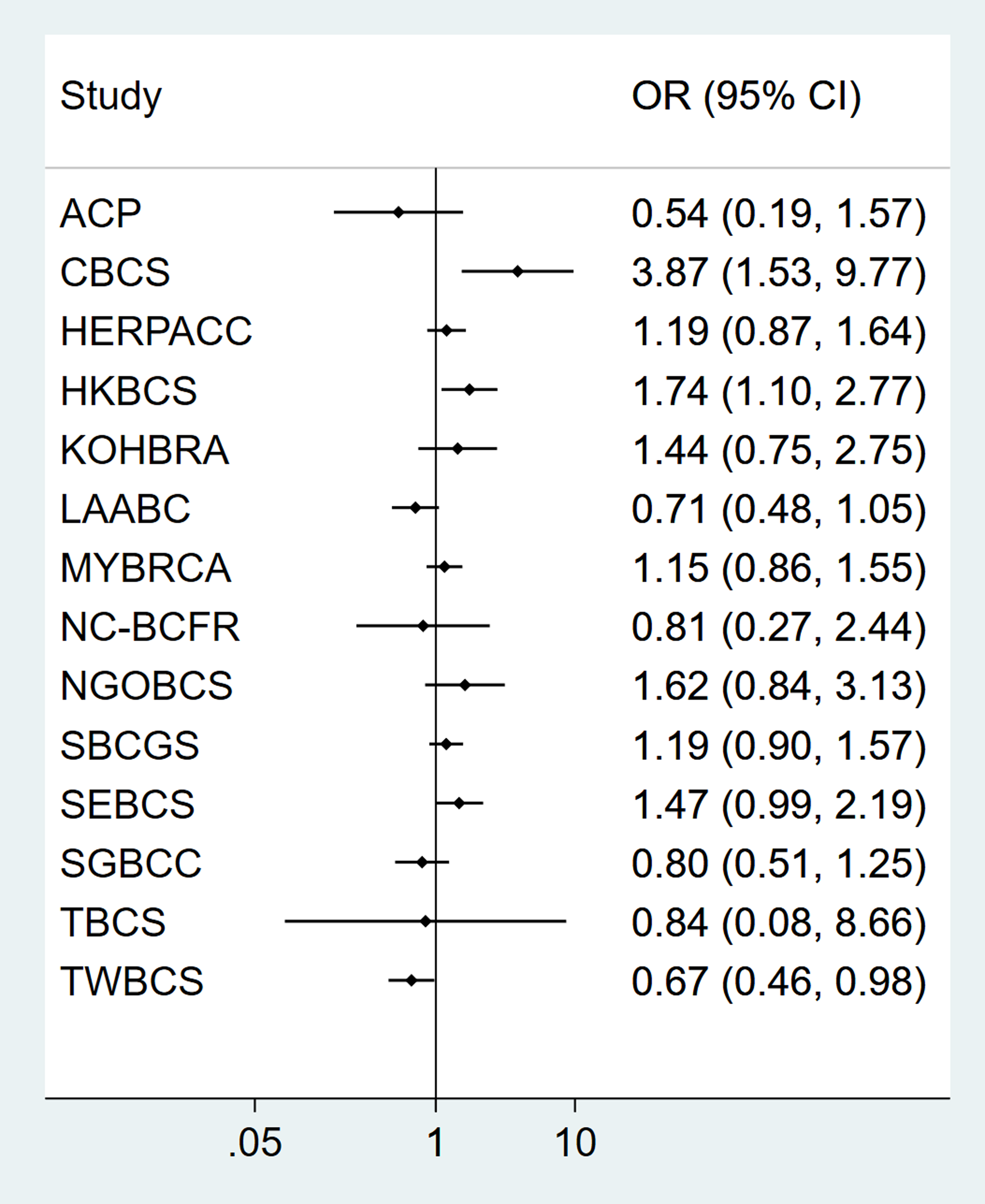

Supplement: Supplementary file 2 [file MGG3-7-e707-s002.tif]
