# Supplementary material for: The functional ALDH2 polymorphism is associated with breast cancer risk: A pooled analysis from the Breast Cancer Association Consortium
Source: Mol Genet Genomic Med. 2019 May 7;7(6):e707. doi: 10.1002/mgg3.707 (PMC6565553; doi:10.1002/mgg3.707)
Supplement: Supplementary file 3 [file MGG3-7-e707-s003.docx]

**Table S1.** Associations between *ALDH2* polymorphism and breast cancer risk by menopausal status

|  |  | **Premenopausal** | | |  | **Postmenopausal** | | |  |  |  |
| --- | --- | --- | --- | --- | --- | --- | --- | --- | --- | --- | --- |
|  |  | **ALDH2 genotype** | | |  | **ALDH2 genotype** | | |  | **P for heterogeneity^b^** | |
|  |  | **Glu/Glu** | **Glu/Lys** | **Lys/Lys** |  | **Glu/Glu** | **Glu/Lys** | **Lys/Lys** |  | **For Glu/Lys** | **For Lys/Lys** |
| **Overall** |  |  |  |  |  |  |  |  |  |  |  |
| Cases /Controls |  | 3,980/4,130 | 2,032/2,052 | 365/309 |  | 3,801/3,908 | 2,038/2,123 | 379/362 |  |  |  |
| OR (95% CI)^a^ |  | 1 (ref.) | 1.04 (0.96-1.13, p=0.295) | 1.21 (1.03-1.43, p=0.024) |  | 1 (ref.) | 0.99 (0.91-1.07, p=0.785) | 1.08 (0.92-1.27, p=0.345) |  | 0.406 | 0.327 |
|  |  |  |  |  |  |  |  |  |  |  |  |
| **ER status** |  |  |  |  |  |  |  |  |  |  |  |
| **Positive** |  |  |  |  |  |  |  |  |  |  |  |
| Cases /Controls |  | 2,416/4,130 | 1,277/2,052 | 243/309 |  | 2,220/3,908 | 1,254/2,123 | 238/362 |  |  |  |
| OR (95% CI)^a^ |  | 1 (ref.) | 1.02 (0.93-1.12, p=0.630) | **1.25 (1.04-1.50, p=0.018)** |  | 1 (ref.) | 1.03 (0.94-1.13, p=0.542) | 1.13 (0.94-1.36, p=0.181) |  | 0.883 | 0.447 |
|  |  |  |  |  |  |  |  |  |  |  |  |
| **Negative** |  |  |  |  |  |  |  |  |  |  |  |
| Cases /Controls |  | 1,147/4,130 | 579/2,052 | 90/309 |  | 1,174/3,908 | 608/2,123 | 103/362 |  |  |  |
| OR (95% CI)^a^ |  | 1 (ref.) | 1.05 (0.93-1.18, p=0.408) | 1.05 (0.81-1.35, p=0.730) |  | 1 (ref.) | 1.01 (0.90-1.16, p=0.860) | 1.00 (0.79-1.27, p=0.987) |  | 0.649 | 0.785 |
|  |  |  |  |  |  |  |  |  |  |  |  |
| **PR status** |  |  |  |  |  |  |  |  |  |  |  |
| **Positive** |  |  |  |  |  |  |  |  |  |  |  |
| Cases /Controls |  | 2,116/4,130 | 1,095/2,052 | 219/309 |  | 2,116/3,908 | 1,0952/2,123 | 219/362 |  |  |  |
| OR (95% CI)^a^ |  | 1 (ref.) | 0.97 (0.88-1.06, p=0.483) | **1.23 (1.02-1.49, p=0.031)** |  | 1 (ref.) | 1.01 (0.91-1.11, p=0.876) | 1.08 (0.89-1.31, p=0.441) |  | 0.733 | 0.669 |
|  |  |  |  |  |  |  |  |  |  |  |  |
| **Negative** |  |  |  |  |  |  |  |  |  |  |  |
| Cases /Controls |  | 1,106/4,130 | 571/2,052 | 87/309 |  | 1,106/3,908 | 571/2,123 | 87/362 |  |  |  |
| OR (95% CI)^a^ |  | 1 (ref.) | 1.04 (0.92-1.17, p=0.531) | 1.03 (0.80-1.35, p=0.767) |  | 1 (ref.) | 1.01 (0.90-1.13, p=0.845) | 1.11 (0.88-1.38, p=0.380) |  | 0.733 | 0.669 |
|  |  |  |  |  |  |  |  |  |  |  |  |
| **HER2 status** |  |  |  |  |  |  |  |  |  |  |  |
| **Positive** |  |  |  |  |  |  |  |  |  |  |  |
| Cases /Controls |  | 1,090/4,130 | 527/2,052 | 76/309 |  | 1,090/3,908 | 527/2,123 | 76/362 |  |  |  |
| OR (95% CI)^a^ |  | 1 (ref.) | 1.02 (0.89-1.17, p=0.761) | 1.07 (0.80-1.43, p=0.658) |  | 1 (ref.) | 1.01 (0.87-1.17, p=0.894) | 1.24 (0.93-1.64, p=0.138) |  | 0.924 | 0.48 |
|  |  |  |  |  |  |  |  |  |  |  |  |
| **Negative** |  |  |  |  |  |  |  |  |  |  |  |
| Cases /Controls |  | 1,363/4,130 | 645/2,052 | 120/309 |  | 1,363/3,908 | 645/2,123 | 120/362 |  |  |  |
| OR (95% CI)^a^ |  | 1 (ref.) | 0.96 (0.85-1.08, p=0.497) | 1.21 (0.95-1.53, p=0.122) |  | 1 (ref.) | 1.10 (0.97-1.24, p=0.126) | 1.27 (1.01-1.60, p=0.041) |  | 0.127 | 0.776 |
|  |  |  |  |  |  |  |  |  |  |  |  |
| **Luminal** |  |  |  |  |  |  |  |  |  |  |  |
| Cases /Controls |  | 1,060/4130 | 477/2052 | 97/309 |  | 890/3908 | 502/2123 | 101/362 |  |  |  |
| OR (95% CI)^a^ |  | 1 (ref.) | 0.92 (0.78-1.09, p=0.323) | 1.26 (0.98-1.63, p=0.074) |  | 1 (ref.) | 1.03 (0.88-1.22, p=0.691) | 1.31 (1.02-1.69, p=0.032) |  | 0.332 | 0.830 |
|  |  |  |  |  |  |  |  |  |  |  |  |
| **Triple positive** |  |  |  |  |  |  |  |  |  |  |  |
| Cases /Controls |  | 721/4130 | 346/2052 | 48/309 |  | 721/4130 | 346/2052 | 48/309 |  |  |  |
| OR (95% CI)^a^ |  | 1 (ref.) | 1.02 (0.87-1.19, p=0.800) | 1.08 (0.77-1.53, p=0.654) |  | 1 (ref.) | 1.07 (0.89-1.30, p=0.470) | 1.34 (0.94-1.91, p=0.103) |  | 0.700 | 0.388 |
|  |  |  |  |  |  |  |  |  |  |  |  |
| **HER2 enrich** |  |  |  |  |  |  |  |  |  |  |  |
| Cases /Controls |  | 342/4130 | 158/2052 | 26/309 |  | 342/4130 | 158/2052 | 26/309 |  |  |  |
| OR (95% CI)^a^ |  | 1 (ref.) | 0.95 (0.77-1.18, p=0.662) | 1.13 (0.73-1.76, p=0.589) |  | 1 (ref.) | 0.97 (0.79-1.19, p=0.802) | 1.14 (0.75-1.72, p=0.544) |  | 0.889 | 0.977 |
|  |  |  |  |  |  |  |  |  |  |  |  |
| **Triple negative** |  |  |  |  |  |  |  |  |  |  |  |
| Cases /Controls |  | 292/4130 | 165/2052 | 23/309 |  | 292/4130 | 165/2052 | 23/309 |  |  |  |
| OR (95% CI)^a^ |  | 1 (ref.) | 1.20 (0.98-1.48, p=0.081) | 1.13 (0.71-1.79, p=0.597) |  | 1 (ref.) | 1.08 (0.86-1.34, p=0.521) | 1.10 (0.70-1.74, p=0.674) |  | 0.816 | 0.943 |
|  |  |  |  |  |  |  |  |  |  |  |  |

^a^ ORs were adjusted for age (continuous), Asian principal components and study sites.

^b^ heterogeneity by menopausal status.

Abbreviation : OR, odds ratio; ER, estrogen receptor; PR, progesterone receptor; HER2, human epidermal growth factor receptor 2
