# Supplementary material for: The functional ALDH2 polymorphism is associated with breast cancer risk: A pooled analysis from the Breast Cancer Association Consortium
Source: Mol Genet Genomic Med. 2019 May 7;7(6):e707. doi: 10.1002/mgg3.707 (PMC6565553; doi:10.1002/mgg3.707)
Supplement: Supplementary file 4 [file MGG3-7-e707-s004.docx]

**Table S2.** Associations between ALDH2 polymorphism and breast cancer risk by random effects meta-analyses

|  |  | **ALDH2 genotype** | | | **P for heterogeneity between tumor characteristics** | |
| --- | --- | --- | --- | --- | --- | --- |
|  |  | **Glu/Glu** | **Glu/Lys** | **Lys/Lys** | **For Glu/Lys** | **For Lys/Lys** |
| **Overall** |  |  |  |  |  |  |
| Cases /Controls |  | 7,781/8,038 | 4,070/4,175 | 744/671 |  |  |
| OR (95% CI)^a^ |  | 1 (ref.) | 1.00 (0.94-1.06, p=0.898) | 1.12 (0.98-1.25, p=0.098) |  |  |
|  |  |  |  |  |  |  |
| **ER status** |  |  |  |  |  |  |
| **Positive** |  |  |  |  |  |  |
| Cases /Controls |  | 4,636/8,038 | 2,531/4,175 | 481/671 |  |  |
| OR (95% CI)^a^ |  | 1 (ref.) | 0.99 (0.92-1.06, p=0.615) | 1.15 (1.01-1.31, p=0.005) | 0.849 | 0.435 |
|  |  |  |  |  |  |  |
| **Negative** |  |  |  |  |  |  |
| Cases /Controls |  | 2,321/8,038 | 1,187/4,175 | 193/671 |  |  |
| OR (95% CI)^a^ |  | 1 (ref.) | 1.01 (0.93-1.11, p=0.679) | 1.05 (0.87-1.260, p=0.506) |  |  |
|  |  |  |  |  |  |  |
| **PR status** |  |  |  |  |  |  |
| **Positive** |  |  |  |  |  |  |
| Cases /Controls |  | 3,842/8,038 | 2,066/4,175 | 400/671 |  |  |
| OR (95% CI)^a^ |  | 1 (ref.) | 0.97 (0.90-1.04, p=0.227) | 1.17 (1.01-1.34, p=0.003) | 0.407 | 0.551 |
|  |  |  |  |  |  |  |
| **Negative** |  |  |  |  |  |  |
| Cases /Controls |  | 2,333/8,038 | 1,238/4,175 | 205/671 |  |  |
| OR (95% CI)^a^ |  | 1 (ref.) | 1.02 (0.93-1.10, p=0.633) | 1.09 (0.91-1.30, p=0.210) |  |  |
|  |  |  |  |  |  |  |
| **HER2 status** |  |  |  |  |  |  |
| **Positive** |  |  |  |  |  |  |
| Cases /Controls |  | 1,961/8,038 | 940/4,175 | 153/671 |  |  |
| OR (95% CI)^a^ |  | 1 (ref.) | 1.01 (0.91-1.13, p=0.708) | 1.17 (0.94-1.46, p=0.051) | 1.000 | 0.610 |
|  |  |  |  |  |  |  |
| **Negative** |  |  |  |  |  |  |
| Cases /Controls |  | 2,521/7,841 | 1,287/4,175 | 246/671 |  |  |
| OR (95% CI)^a^ |  | 1 (ref.) | 1.01 (0.92-1.10, p=0.781) | 1.26 (1.05-1.50, p<0.001) |  |  |

^a^ ORs were adjusted for age (continuous), Asian principal components and study site.

Abbreviation : ER, estrogen receptor; PR, progesterone receptor; HER2, human epidermal growth factor receptor 2
