# Supplementary material for: The functional ALDH2 polymorphism is associated with breast cancer risk: A pooled analysis from the Breast Cancer Association Consortium
Source: Mol Genet Genomic Med. 2019 May 7;7(6):e707. doi: 10.1002/mgg3.707 (PMC6565553; doi:10.1002/mgg3.707)
Supplement: Supplementary file 5 [file MGG3-7-e707-s005.docx]

**Table S3.** Association between *ALDH2* polymorphism and breast cancer risk according to alcohol intake

|  |  | **ALDH2 genotype** | | | |  | **P for interaction^b^** | |
| --- | --- | --- | --- | --- | --- | --- | --- | --- |
|  |  | **Glu/Glu** | **Glu/Lys** | **Lys/Lys** | |  |  |  |
| **Non-drinker** |  |  |  |  | |  |  | |
| Cases /Controls |  | 828/1,040 | 745/1,076 | 173/232 | |  |  | |
| OR (95% CI)^a^ |  | 1 (ref.) | 0.91 (0.79-1.06, p=0.219) | 0.90 (0.72-1.14, p=0.393) | |  | 0.537 | |
|  |  |  |  |  | |  |  | |
| **Any drinker** |  |  |  |  | |  |  | |
| Cases /Controls |  | 897/1,010 | 203/320 | 3/2 | |  |  | |
| OR (95% CI)^a^ |  | 1 (ref.) | 0.86 (0.69-1.07, p=0.172) | NE | |  |  | |
|  |  |  |  |  | |  |  | |
| **<15 g ethanol/day** |  |  |  |  | |  |  | |
| Cases /Controls |  | 719/774 | 173/276 | 3/2 | |  |  | |
| OR (95% CI)^a^ |  | 1 (ref.) | 0.84 (0.66-1.05, p=0.128) | NE | |  |  | |
|  |  |  |  |  | |  |  | |
| **≥15 g ethanol/day** |  |  |  |  | |  |  | |
| Cases /Controls |  | 178/236 | 30/44 | 0/0 | |  |  | |
| OR (95% CI)^a^ |  | 1 (ref.) | 1.02 (0.61-1.73, p=0.933) | NE | |  |  | |
|  |  |  |  |  | |  |  | |
| **<15 g ethanol/day** |  |  |  |  |  |  | |  |
| Cases /Controls |  | 719/774 | 173/276 | 3/2 |  |  | |  |
| OR (95% CI)^a^ |  | 1 (ref.) | 0.83 (0.66-1.04, p=0.111) | NE |  |  | |  |
|  |  |  |  |  |  |  | |  |
| **15-30 g ethanol/day** |  |  |  |  |  |  | |  |
| Cases /Controls |  | 101/149 | 14/26 | 0/0 |  |  | |  |
| OR (95% CI)^a^ |  | 1 (ref.) | 1.01 (0.45-2.28, p=0.660) | NE |  |  | |  |
|  |  |  |  |  |  |  | |  |
| **≥30 g ethanol/day** |  |  |  |  |  |  | |  |
| Cases /Controls |  | 77/87 | 16/18 | 0/0 |  |  | |  |
| OR (95% CI)^a^ |  | 1 (ref.) | 1.28 (0.49-3.33, p=0.611) | NE |  |  | |  |
|  |  |  |  |  | |  |  | |

^a^ ORs were adjusted for age, Asian principal components and study site.

^b^ Interaction between ALDH2 genotype (Glu/Glu vs Glu/Lys+Lys/Lys) and alcohol intake (non vs any).

Abbreviation : NE, not estimated due to small sample size
